# Study of Mechanical Properties of PHBHV/Miscanthus Green Composites Using Combined Experimental and Micromechanical Approaches

**DOI:** 10.3390/polym13162650

**Published:** 2021-08-10

**Authors:** Thibault Lemaire, Erica Gea Rodi, Valérie Langlois, Estelle Renard, Vittorio Sansalone

**Affiliations:** 1 MSME, UMR 8208, Univ. Paris Est Creteil, Univ. Gustave Eiffel, CNRS, 94010 Creteil, France; egea.rodi@gmail.com (E.G.R.); vittorio.sansalone@u-pec.fr (V.S.); 2 ICMPE, CNRS UMR 7182, Univ. Paris Est Creteil, 94320 Thiais, France; langlois@u-pec.fr (V.L.); renard@icmpe.cnrs.fr (E.R.)

**Keywords:** biocomposite, bio-sourced products, mechanical properties, micro-mechanics, mechanical testing

## Abstract

In recent years the interest in the realization of green wood plastic composites (GWPC) materials has increased due to the necessity of reducing the proliferation of synthetic plastics. In this work, we study a specific class of GWPCs from its synthesis to the characterization of its mechanical properties. These properties are related to the underlying microstructure using both experimental and modeling approaches. Different contents of Miscanthus giganteus fibers, at 5, 10, 20, 30 weight percent’s, were thus combined to a microbial matrix, namely poly (3-hydroxybutyrate)-co-poly(3-hydroxyvalerate) (PHBHV). The samples were manufactured by extrusion and injection molding processing. The obtained samples were then characterized by cyclic-tensile tests, pycnometer testing, differential scanning calorimetry, Fourier transform infrared spectroscopy, X-ray diffraction, and microscopy. The possible effect of the fabrication process on the fibers size is also checked. In parallel, the measured properties of the biocomposite were also estimated using a Mori–Tanaka approach to derive the effective behavior of the composite. As expected, the addition of reinforcement to the polymer matrix results in composites with higher Young moduli on the one hand, and lower failure strains and tensile strengths on the other hand (tensile modulus was increased by 100% and tensile strength decreased by 23% when reinforced with 30 wt % of Miscanthus fibers).

## 1. Introduction

In recent years, the market for wood plastic composites (WPCs) has grown exponentially thanks to the possibility to assure durability to the manufactured products without the use of toxic chemical agents or harmful fibers [1]. The different applications for these materials, such as in building and construction field or automotive, make this market highly fragmented, but at the same time very attractive for many research groups and industries. Today, most of these composites are constituted by matrices derived from oil such as polyethylene (PE) or polypropylene (PP), in which a small amount of process and property modifiers are added though extrusion or injection processes to enhance outdoor applications [2]. Even though natural cellulosic fibers have been successfully used with petroleum-derived polymers, the environmental benefits of natural fiber composites can be enhanced considerably if biodegradable polymers are used [3]. These biocomposites can be easily disposed of or composted at the end of their life without harming the environment, which is not possible with synthetic fiber-based polymer composites. In fact, the addition of natural fillers increases the renewable content in the final product without changing the biodegradability of the continuous phase and also allows the use of agroforestry and fruit wastes, marking an important transition of the economy from a linear to circular model [4,5].

Poly(3-hydroxyalkanoates) (PHAs) are a class of natural biodegradable polyesters accumulated by many bacteria as carbon and energy supply when an essential nutrient is limited [6,7]. Using various substrates, a wide variety of PHAs can be synthesized, differing notably in the length of their side chains [6,8]. Two types of PHAs can be distinguished: (i) short-chain-length PHAs, or scl-PHAs, possessing alkyl side chains with up to two carbon atoms, as the widely used poly(3-hydroxybutyrate-co-3-hydroxyvalerate) PHBHV, for example, is considered hereafter; (ii) medium-chain-length PHAs, or mcl-PHAs, with at least three carbon atoms in their side chains. PHAs have been suggested as green substitutes for conventional plastics, due to their synthesis from renewable resources and their biodegradation by enzymatic action [9]. Owing to their biocompatibility and biodegradability, PHAs proved to be good candidates for biomedical applications, including in the design of devices, biodegradable drug carriers, and tissue engineering (TE) scaffolds [10,11,12,13]. They have also been combined with natural cellulosic fibers such as hemp jute flax carnauba fibers, miscanthus, bamboo [14,15,16,17], pineapple fibers [18], recycled wood fiber [19], and cellulose nanowhisker [20,21] to prepare biocomposites.

Among all the existing PHAs, poly(3-hydroxybutyrate) (PHB) is certainly one of the most important. It possesses a melting point close to that of polypropylene, better oxygen barrier property, and similar mechanical properties [9]. However, its brittleness and narrow processing temperature window limit its application. To overcome the inferior properties of PHB, a variety of copolymers were synthesized by bioconversion, such as the poly(3-hydroxybutyrate-co-3-hydroxyvalerate) (PHBHV) with 12% of valerate units was chosen to realize composite materials. Among the different vegetable fibers that can be used, we chose Miscanthus (*Miscanthus giganteus*). This is a perennial crop, highly productive with a very efficient nitrogen-recycling system and a very interesting energy balance due to the absence of nitrogen [22] and recently used in composites [23,24,25,26]. A well-known phenomenon in these materials is the incompatibility between fibers and matrix caused by the hydrophobicity of the polymer and the hydrophilic nature of the fibers. This effect has serious consequences on the mechanical seal of the final material which is why compatibilizer agents, additives, or surface treatments that improve the cohesion at the matrix/fiber interface are often necessary [27,28,29,30,31,32,33]. Thus, we recently showed the possibility to improve the adhesion between poly(caprolactone) and vegetal fibers using photoactivated grafting of a bounding agent [34,35]. In this update paper, by working with unfunctionalized materials, we intend to present a complete study of PHBHV/Miscanthus composites from their synthesis to their testing and in silico characterization. More specifically, this paper is not an increment in the enhancement of the fiber compatibility with the matrix, but a necessary work providing a starting reference that combines an extensive experimental characterization of PHBHV/Miscanthus composites and its associated modelling tool.

Considering different content of Miscanthus fibers, the purpose of this study is to propose a rigorous method to produce these composites and then determine a reference protocol to depict their physical and mechanical properties. This characterization is performed using a twofold approach since, in addition to classical mechanical and physical testing, a micromechanical approach taking into account the geometry, volume fraction, and organization of the fibers in the matrix, is carried out to estimate the effective mechanical properties of the composite.

At this stage, the in silico methods are validated using non-functionalized media. This is necessary before introducing interfacial terms in the model to represent enhanced behaviors of functionalized composites. Then, in a further step, this modelling approach will be useful to derive a model-driven optimization of the composite for enhanced synthesis such as the one proposed by Rodi et al. [17]. After having briefly presented the composites processing, the different experimental and numerical characterization tools are introduced in the materials and methods section. These characterizations consist in scanning electron microscopy, mechanical testing, fibers size comparison, pycnometer testing, differential scanning calorimetry, Fourier transform infrared spectroscopy, and X-ray diffraction. Then the results are presented and finally discussed.

## 2. Materials and Methods

### 2.1. Materials

Poly(3-hydroxybutyrate-co-3-hydroxyvalerate) (PHB_88_HV_12_), containing 12% of valerate, was purchased from Goodfellow, Lille, France, in a pelletized form. *Miscanthus giganteus* (MIS) fibers were provided by Miscanplus, Digny, France. MIS fibers came from a 2014 spring crop roughly chopped and subsequently milled until fiber length ranged between 1 and 5 mm.

### 2.2. Composite Processing

Thermogravimetric analysis revealed that the MIS has ambient moisture of 5%. For this reason, prior to processing, both PHBHV and Miscanthus were dried in a conventional oven at 80 °C for 5 h in order to remove any moisture. In order to prevent the absorption of moisture, products were stored in a desiccator containing K_2_HPO_4_ prior to processing. To investigate the effect of the fiber content on composite mechanical properties, the following nominal values were investigated: 0, 5, 10, 20, and 30 wt %. The nominal mass content corresponds to the mass content of MIS at the beginning of the composite processing. As shown later in this paper (see Section 3.4), the fiber mass content of the final product may be slightly lower. Notwithstanding this observation, the classical use of nominal contents is adopted hereafter to present the results.

According to the nominal mass content of each phase, PHBHV and MIS were mixed together in a lab-scale twin-screw extruder (Minilab Thermo Scientific Haake, Waltham, MA, USA). The experiments were performed at 160 °C (T_E_) with a screw speed of 60 rpm (n). The retention time for the pure matrix was 1 min; this time was increased to 2 min in order to fully disperse the fibers into the matrix. After recirculation, the extruded molten material was transferred by means of a preheated piston-cylinder assembly and was shot in the micro-injection unit (MiniJet Thermo Scientific Haake, Waltham, MA, USA) at the injection pressure (P_I_) for 30 s. A maintenance pressure (P_M_), lower than that used during the phase of injection, was applied for another 30 s. The collector and the mold temperatures were set at 165 °C (T_I_) and 45 °C (T_m_), respectively. Parameters during the phase of injection of the material were adjusted according to the increase in the polymer melt viscosity with the fiber content. Some of the final optimized parameters used for the entire process are resumed in Table 1.

### 2.3. Materials Characterization

#### 2.3.1. Scanning Electron Microscope (SEM)

SEM observations were performed using a Merlin Carl Zeiss, Marly le Roi, France, scanning electron microscope. Prior to observation, the cross sections of specimens at different fiber content were sputter-coated with a thin layer of palladium in a Cressington (Watford, UK) 208 HR sputter-coater. Images were recorded with an acceleration voltage of 10 keV and at different magnifications.

#### 2.3.2. Mechanical Properties

The mechanical properties of the composites were evaluated using an Instron 5965 Universal Testing Machine, Norwood, MA, USA, equipped with a cell load of 100 N. All specimens presented standard dimensions according to ASTM638 (West Conshohocken, PA, USA). Two types of tests were made. On the one hand, a simple traction test was set up at a rate of 5 mm/min in order to evaluate the mechanical behavior of the composites, typically its failure strain and tensile strength. Five samples were tested for each fiber content value to obtain standard deviation values. These tests were performed 2 days after the day of realization of the biocomposites. On the other hand, a cyclic traction test was set up to evaluate the Young modulus. Ten specimens for each fiber content value were tested in this setup. Prior to testing, specimens were stored for 8 days at 23 °C. The cyclic traction test was set up with increasing values of the maximum load applied by the testing machine from one cycle to another. The initial maximum state stress was set to 3 N and the final one to 10 N with an increment of 1 N from one cycle to another (i.e., 8 cycles overall). The lower and upper limits were related to the sensitivity of the experimental device threshold and to the yield stress of the PHBHV, respectively. We, moreover, checked a posteriori that the cyclic loading curves remain in the elastic domain. All cycles were made at a constant speed of 0.05 N/s.

#### 2.3.3. Fiber-Size Distribution

After processing, specimens with different fiber content were solubilized three times in dichloromethane during 30 min. After filtration, the collected Miscanthus fibers were observed using a 3B Scientific Physics, Bartenheim, France, microscope at a magnification of 4×. A series of 10 observations was made per specimen.

#### 2.3.4. Density Measurements

Average density of pure matrix and composites were evaluated using a helium AccuPyc 1330 Micromeritics, Norcross, GA, USA, pycnometer on around 40 mg of mass taken from the central section of the specimens used for tensile tests. The density calculated with this method was compared with that calculated using the ratio between the mass and the volume of the specimens.

#### 2.3.5. Thermal Analyses

Differential scanning calorimetry experiments were performed on a PerkinElmer Diamond DSC Apparatus, Haguenau, France. Sample of around 10 mg sealed in aluminum pans were initially heated from −60 °C to 200 °C at 20 °C/min, cooled down rapidly, and then reheated in the same conditions used in the first heating run. Melting point (T_M_) and melting enthalpy (ΔH_M_) were determined during the first heating. The degree of crystallization (Xc) was then calculated using the equation

Xc (%) = 100 × ΔH_M_/(ΔH_0_ × W) (1)

where ΔH_0_ corresponds to the melting enthalpy of a 100% crystalline PHBHV (146 J/g) and W is the polymer fraction present in the composite.

#### 2.3.6. Fourier Transform Infrared Spectroscopy (FTIR)

Different biocomposites were prepared, varying the content of fibers (5 and 20 wt %) and the length of fibers (1000 and 45 µm). The specimens of PHBHV/MIS composites were then solubilized in dichloromethane in order to separate fibers from matrix. The collected fibers were extracted 3 times in 100 mL of dichloromethane at 54 °C, stirring for 30 min at 200 rpm. Fibers were then dried before analysis. Infrared spectra of the extracted fibers were recorded using a TENSOR27 Brucker (Champs sur Marne, France) apparatus equipped with an attenuated internal reflection accessory using a diamond crystal (Digi Tech DLATGS Detector, 32 scans, 4 cm^−1^) in the range 500–4000 cm^−1^. These spectra were then compared with that of raw Miscanthus fibers to assess the natural grafting of PHBHV on the MIS through the ratio R calculated as follows:

(2) $$R=\frac{I_{{1726\mathrm{cm}}^{-1}}}{I_{{1604\mathrm{cm}}^{-1}}}$$

where I_1726_ corresponds to the intensity of carbonyl group of PHBHV and I_1604_ corresponds to the intensity of the esters present in the lignin structure.

FTIR is also an useful analysis tool to evaluate the crystallinity of the PHBHV after processing. For instance, the band at 1726 cm^−1^ is representative of the C=O stretch present in the highly crystalline structure of the matrix, while the small shoulder at 1740 cm^−1^ represents the same stretch in the amorphous region. Typically, the absorption bands at 1720, 1276, 1225, and 980 cm^−1^ are representatives of the crystalline regions, whereas the bands at 1740, 1452, and 1176 cm^−1^ are representatives of the amorphous ones [36,37]. In particular, the band around 1378 cm^−1^ corresponds to the symmetrical wagging of the CH3 groups and that at 1452 cm^−1^ to the asymmetric deformation of methylene groups. These bands are considered as insensitive to crystallinity and they can be good candidates to evaluate the crystallinity degree. Hereafter, the band at 1452 cm^−1^ is thus considered in addition to the one at 1225 cm^−1^; the latter corresponding to the C-O-C stretching which is representative of the crystalline regions. Thus, we calculate the crystallinity index C_I_

(3) $$\mathrm{CI}=\frac{I_{{1225\mathrm{cm}}^{-1}}}{I_{{1452\mathrm{cm}}^{-1}}}$$

where I_1225_ is assigned to the C-O-C stretching mode of the crystalline parts, and I_1452_ corresponds to the asymmetric deformation of the methylene groups (insensitive to crystallinity). This index provides qualitative information about all changes that may occur in the crystalline structure of the matrix.

#### 2.3.7. X-ray Diffraction

Structural characterizations of Miscanthus fibers and PHBHV/MIS composites were determined by X-ray diffraction (XRD) using a D8 advance Bruker (Champs sur Marne, France) diffractometer operating at 40 kV and 40 mA with a CuK α radiation. The whole area investigated was in the range 2θ ≈ 5–40° at a scanning rate of 0.2°/min.

### 2.4. Modelling

Among the several methods predicting the elastic properties of fiber-reinforced composites, the rule of mixtures (ROM) is probably the quickest and easiest one. Using the elastic moduli $E_{F}$ and $E_{M}$ of the fiber and matrix phases, and the volume fraction of the fibers $\phi_{F}$ , the effective Young modulus of the composite reads:

(4) $$E_{C}=\phi_{F}E_{F}+(1-\phi_{F})E_{M}$$

**Figure 1:**
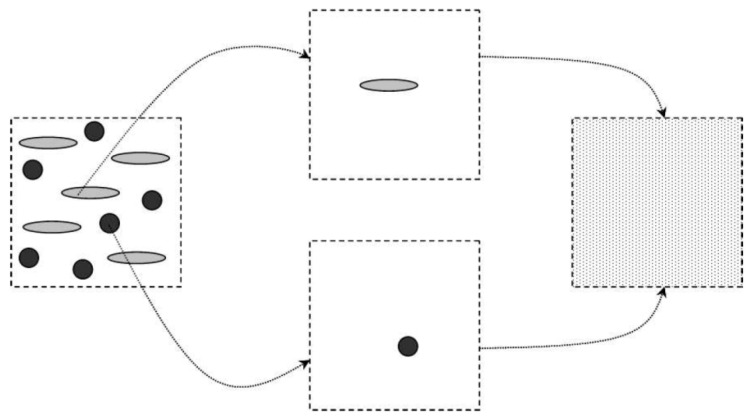
Schematic of the Mori–Tanaka model. On the left: actual, heterogeneous material; on the right: effective, homogeneous material. The intermediate step represents the homogenization procedure where individual inclusions are considered as embedded in the matrix phase and contribute to the overall elasticity of the homogenized material.

Much more sophisticated models were also developed to evaluate the effective elastic behavior of the composite using homogenization approaches. Homogenization theories can estimate the effective elastic tensor $C_{\mathrm{hom}}$ of a multiphase material based on information about its microstructural organization. Among others, continuum micromechanics [38,39] proved to be quite useful when dealing with composite materials of matrix-inclusion type [40,41]. Continuum micromechanics use the solution of the matrix-inclusion problem provided by Eshelby in the fifties [42] to estimate the effective elastic tensor $C_{\mathrm{hom}}$ of a multiphase material as [43]:

(5) $$C_{\mathrm{hom}}=\sum_{r}\phi_{r}C_{r}:A_{r}$$

where $\phi_{r}$ , $C_{r}$ , and $A_{r}$ are the volume fraction, (4th-order) elastic tensor and (4th-order) localization tensor of phase *r*, and the sum runs over all the constituent phases. The localization tensor $A_{r}$ accounts for the nature and geometrical organization of the phase *r* within the effective matrix and its expression depends, in general, on the volume fraction and elastic tensor of all the phases. Different estimates of $C_{\mathrm{hom}}$ can be obtained by suitable choices of the effective matrix. As long as one actual phase can be identified as a “matrix” phase, the relevant estimate of $C_{\mathrm{hom}}$ is provided by the Mori–Tanaka model. The idea behind this approach is sketched in Figure 1.

Our case is well described by the Mori–Tanaka model, since MIS fibers (inclusion phase) are disconnected with one another and fully embedded in the PHBHV (matrix phase). Thus, the information required by the model concerns the elastic tensors of the MIS and PHBHV, and the volume fraction and geometrical organization of the MIS fibers. We assumed both MIS and PHBHV to be elastic isotropic materials (see Table 2). The volume fraction of the MIS fibers $\phi_{r}$ was computed based on the measured mass fraction using the procedure outlined in the Appendix B. Eventually, we assumed the MIS fibers to be either cylinder shaped and aligned with the sample main axis (that is to say the extrusion direction) or spherical particles. Note that these two hypotheses lead to homogenized materials which are transversely isotropic and isotropic, respectively.

## 3. Results

### 3.1. Mechanical Properties of Biocomposites

**Figure 2:**
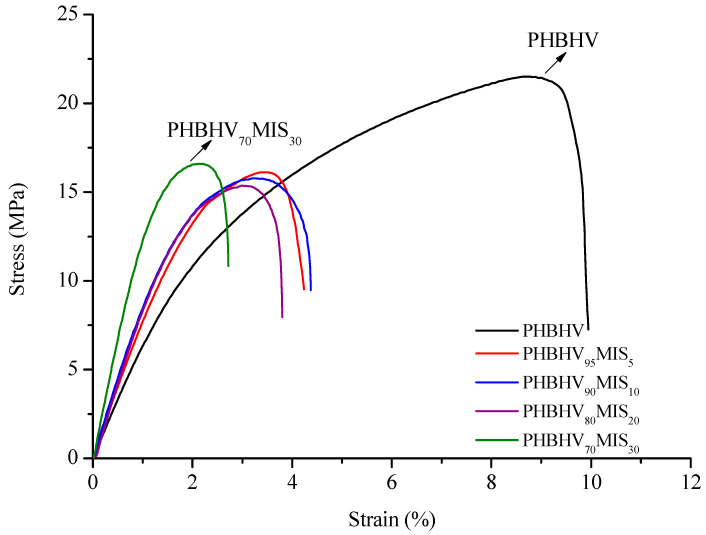
Strain-stress curves for PHBHV/MIS composites (the weight percents contents are indicated).

The tensile modulus, the tensile stress and the ultimate strain of the PHBHV/MIS composites were evaluated using classical tensile tests (see Table 3). For rather low nominal contents of fibers, typically 5, 10, and 20 wt %, the properties change rather slowly. It is necessary to reach 30 wt % of fibers mass content to observe a significant effect of the reinforcement in the matrix. This gradual increase in the tensile modulus is clearly visible comparing the initial slopes of the curves obtained from tensile tests (Figure 2).

In parallel, a series of cyclic loading–unloading tensile tests was performed. First, a pure matrix sample was tested to quantify its damage limit. Through these tests, it was possible to identify the applied force beyond which damage appears in the matrix, which turned out to be about 10 N. This value was used to set up the upper limit of the eight-cycle loading–unloading tensile test performed on all the composite samples (see Section 2.3.2), in order to prevent rupture of the samples. For each test, the slope of each unloading phase was computed and used to obtain the tensile modulus of the composite. This last was definitively corrected using the procedure showed in Appendix B and the final values are showed in Table 4.

**Figure 3:**
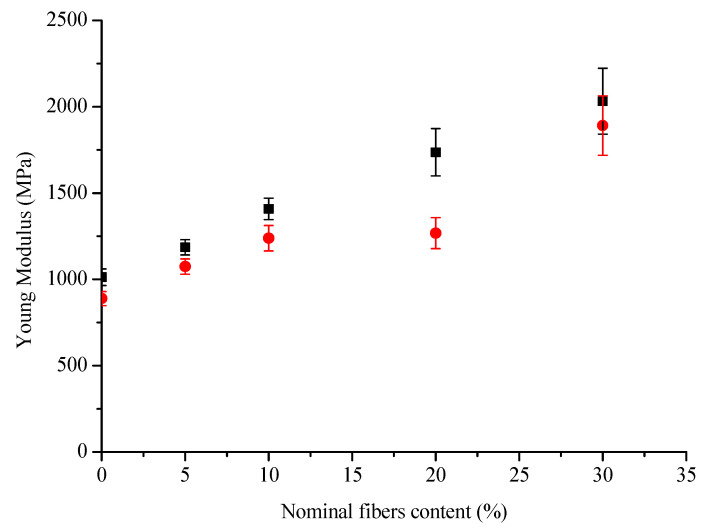
Young Modulus vs Nominal fibers content calculated with the two methods of loading–unloading tests (■) and traction tests (●).

**Figure 4:**
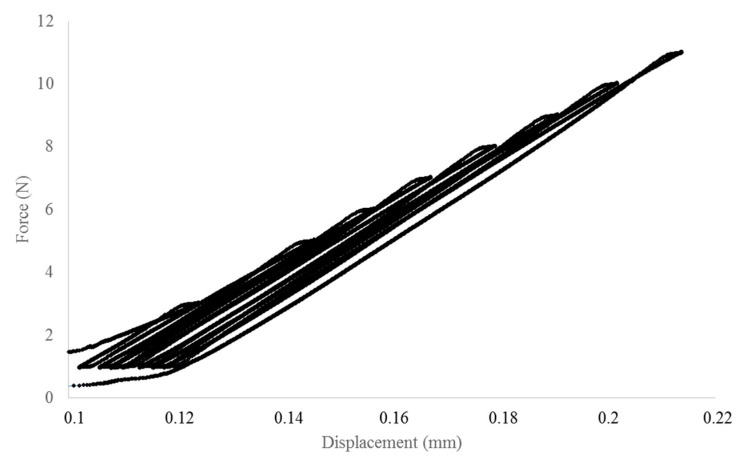
Loading–unloading cycle for a PHBHV_95_MIS_5_ specimen.

The slope, and thus the tensile modulus, increased with increasing fiber content, but also the standard deviations of these values increased. Furthermore, the values obtained with a simple tensile test are lower from those obtained with the cyclic procedure (Figure 3). Another important point of these tests is the difference between the slopes of the curve during the loading and unloading phases, the latter being more important (Figure 4).

### 3.2. Scanning Electron Microscopy (SEM)

**Figure 5:**
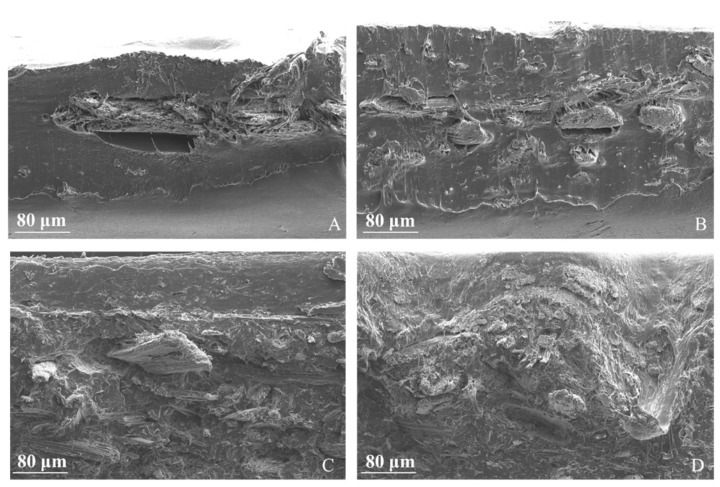
SEM images of the cross section of PHBHV/MIS composites: (**A**) PHBHV_95_MIS_5_; (**B**) PHBHV_90_MIS_10_; (**C**) PHBHV_80_MIS_20_; (**D**) PHBHV_70_MIS_30_.

The cross sections of composites at different content of fibers were characterized by SEM at a magnification of 100; the results are shown in Figure 5. At lower fiber contents, typically at 5 and 10 wt %, the fibers are isolated in the matrix and perfectly identifiable. At higher fiber contents, fibers tend to form aggregates in all the section. In all samples, fibers pull out from the matrix.

### 3.3. Fiber-Size Distribution

Average values of width (D) and length (L) of the fibers and the L/D ratio are reported in Table 5. The fibers aspect ratio decreases when fiber content increases. This indicates that the composite synthesis based on an injection molding processing does damage the fibers for higher content. Indeed, due to the narrow pathway in the injector, it is reasonable to assume that high fiber contents correspond to stronger deterioration of the fibers depending on the injection conditions (see for instance Moritzer et al. [45] or Dupuis et al. [46]). In particular, it could be interesting in a future work to check the effect of annealing after composite processing.

### 3.4. Density of Miscanthus and Composites

In Table 6, the fiber and composites densities are presented, introducing: **ν_MIS_n_**, the nominal mass fraction of Miscanthus; **ν_MIS_m_**, the measured mass fraction; **<ρ_MIS_>**, the average density of Miscanthus; **<ρ_C_calc_>**, the calculated average density of composites; and **<ρ_C_exp_>**, the experimental average density of composites.

First, using the procedure presented in Appendix B to calculate the volumetric fraction of the MIS fibers, the fiber mass $M_{F}$ is obtained and then their density $\rho_{F}$ is determined thanks to Equation (A4). The fiber mass measurements clearly show that discrepancies between the nominal and actual MIS mass contents do exist, the actual value being slightly lower. This trend may be due to a clogging effect at the injection point. Actually, during the injection process, the mixture is cooled quickly from T_I_ = 165° in the collector to T_m_ = 45° in the mold, leading to a progressive clogging at the injection point which increasingly prevents fibers from entering the mold. According to this hypothesis, the bulk of the mold should be richer in fibers than the space near the mold walls, the latter being essentially filled by pure matrix. This phenomenon is clearly visible in the SEM images of Figure 5A,B where there are no fibers in the boundary of the sample. The density of MIS roughly ranged from 0.7 to 1 g/cm^3^ after the injection molding procedure. Note that the MIS fibers extracted from specimens at 5 and 10 wt % from specimens at 20 and 30 wt %, respectively, have similar densities. Secondly, the density of the biocomposites was evaluated through the pycnometer and compared with that obtained from a weighing procedure. Results obtained with the two methods are coherent for 5, 10, and 20 wt %, whereas they differ for the pure PHBHV and the 30 wt % case. We attribute this to a potential misuse of the pycnometer.

### 3.5. Evaluation of the Crystal Structure of PHBHV Matrix

FTIR and XRD investigations were conducted to evaluate the internal crystalline structure of PHBHV and different biocomposites with various contents of fibers (from 5 to 20 wt %) and different lengths of fibers (1000 and 45 µm).

On the one hand, the use of FTIR analysis can provide an indication on the crystalline behavior (see Section 2.3.6). As shown in Table 7, the crystallinity index C_I_ decreased from 1.07 for the neat matrix to 1 for a composite with 20 wt % of long fibers or of 5 wt % of short fibers. Thus, fibers length and content slightly affect the crystalline behavior of the composite. This may, thus, cause differences when measuring the mechanical properties of the different samples.

**Figure 6:**
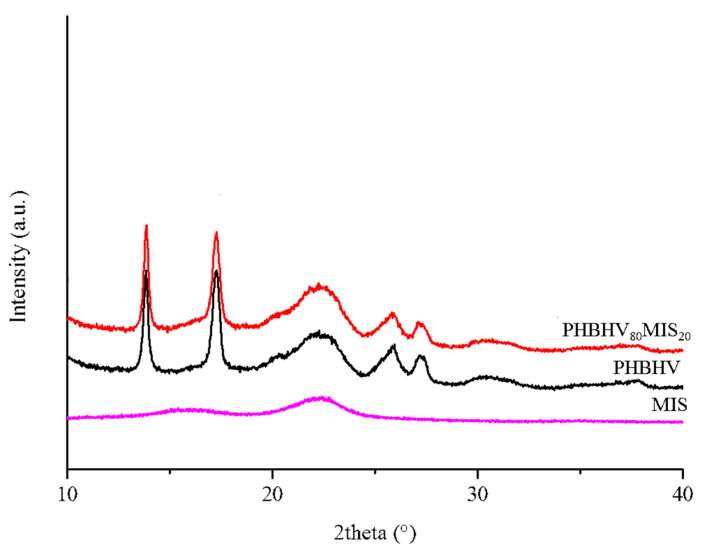
XRD diffractograms of Miscanthus (magenta), PHBHV neat matrix (black), and PHBHV_80_MIS_20_ composites (red).

On the other hand, XRD analysis was conducted on the neat matrix and composites with 20% of long fibers (Figure 6). PHBHV has a semi-crystalline nature with characteristics peaks at 2theta around 13°, 17°, 21°, 22°, 25°, and 27°, corresponding to planes (020), (110), (101), (111), (121), (040), respectively, in the orthorhombic crystalline lattice. The addition of MIS does not alter the basic crystal structure of PHBHV since the reflections are located at the same angle. Moreover, the evaluation of Bravais parameters showed that the lattice volume did not change.

### 3.6. FTIR Analysis to Assess Grafting Efficiency

**Figure 7:**
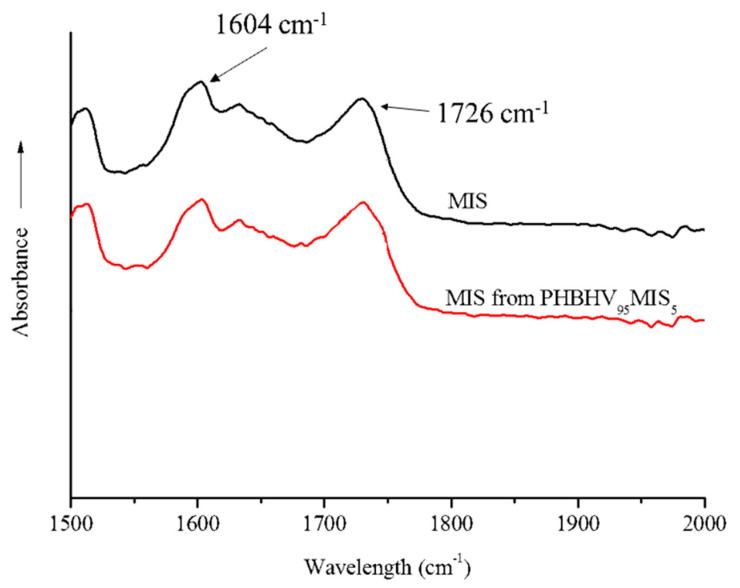
FTIR-ATR spectra of MIS (black curve) and MIS extracted from a composite PHBHV_95_MIS_5_ (red curve).

In parallel, FTIR analysis can also be used to evaluate the quantity of PHBHV chains grafted onto MIS surface. That is why FTIR-ATR spectroscopy analyses were carried out on the fibers extracted from biocomposites PHBHV_95_MIS_5_. In Figure 7, for instance, we present the spectra of raw Miscanthus fibers and of the fibers extracted from biocomposites PHBHV_95_MIS_5_.

The ratio R between the peak at 1726 cm^−1^, corresponding to the carbonyl group of the PHBHV, and the peak at 1604 cm^−1^, corresponding to the esters of lignin, is then evaluated and reported in Table 8. Since this ratio for the composite is the same as the one obtained for the raw Miscanthus, no PHBHV was grafted onto the MIS surface during the process. This suggests a direct prospect of this study by improving the interfacial fibers/matrix properties.

### 3.7. Results of Numerical Simulation

**Figure A1:**
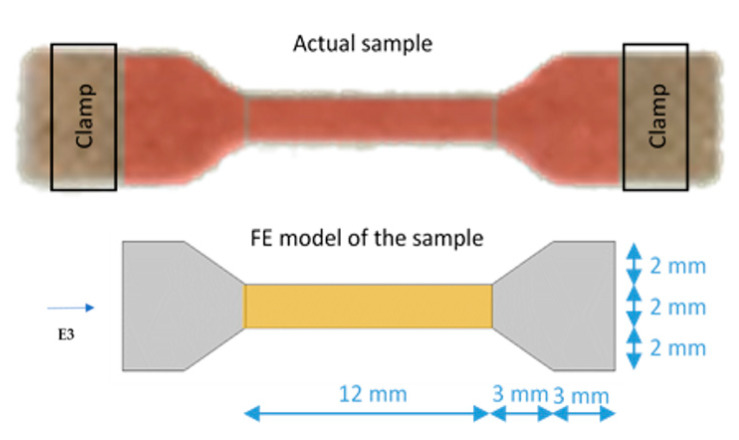
Actual sample (on the top) and 2D FE model of the sample (on the bottom; the “active” part is colored in yellow). The horizontal E3 direction is also presented.

A Mori–Tanaka model was used to estimate the overall homogenized elastic modulus of PHBHV/MIS composites in the direction of application of the stress (horizontal direction E3 in Figure A1). In a first case, we assumed that the fibers had a cylindrical shape and were aligned along the axis of stress, leading to a transversely isotropic effective behavior. In a second case, we considered that fibers had a spherical shape, thus giving an isotropic system. Both models require knowledge of the volumetric fractions of fibers in the biocomposites and the mechanical properties of the constituents. In both models, both matrix and fibers are assumed to be isotropic. Then, their elastic behavior is fully described by their Young modulus and Poisson ratio.

**Figure 8:**
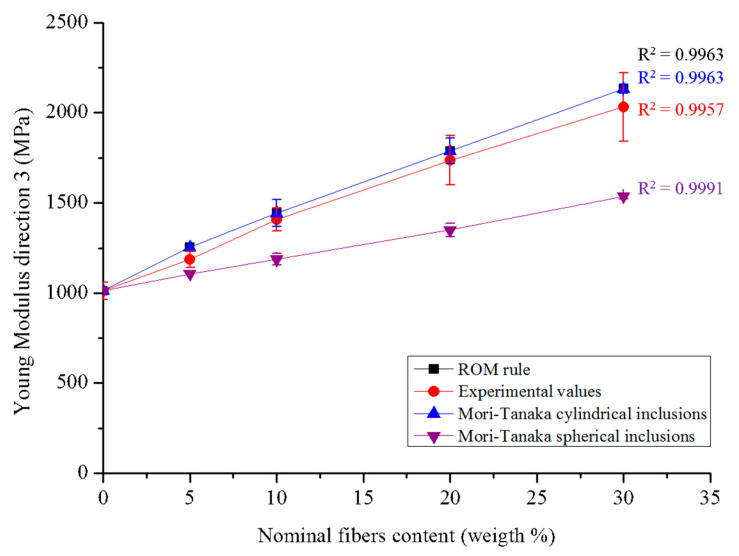
Results of numerical simulation: the values obtained with ROM rule are superposed with those obtained with the Mori–Tanaka with cylindrical inclusions.

The experimental values calculated with the loading–unloading cycles lie between the two simulations, which constitute the upper and lower limits for the elastic modulus E_3_. The model with cylindrical fibers aligned in the direction of the stress seems to get closer to the experimental values than the model with spherical fibers. Moreover, results obtained from the cylindrical fiber model are perfectly overlapped with those obtained using the rule of mixtures (ROM). All these results are shown in Figure 8.

## 4. Discussion

### 4.1. The Mechanical Behavior of PHBHV/MIS Composites

A familiar behavior in composite materials demonstrates an increase in Young’s modulus and a decrease in failure strain and stress when increasing the fiber mass content. This general trend was showed in many previous works [44,47,48,49,50] and it seems to be independent from the nature of the polymer matrix. Biocomposites realized with other bio-based matrix, such as PLA, showed an increase by 20% when reinforced with 20 wt % of Miscanthus fibers and a slight decrease in Young Modulus for percentage over 20 wt %; the latter probably due to the formation of aggregates [26,51]. Comparing the tensile modulus and strength of the polypropylene (PP)-based wood fiber composites with PHBHV–wood fiber composites, the first ones present the higher order of tensile properties [52]. As reported in the literature, the modulus and the strength of a PP composite reinforced with 30 wt % of wood fibers are 3.33 GPa and 27.1 MPa, respectively [53]. In the present work, all the phenomena previously described were highlighted by simple tensile tests (see Table 3) and by loading–unloading tests (see Table 4). For our samples, the tensile modulus was increased by 100% and tensile strength decreased by 23% when reinforced with 30 wt % of Miscanthus fibers as compared to neat PHBHV. These results are in line with the classical observation that stiffer particles normally increase Young’s modulus, but reduces tensile strength of the composite, often due to bad particle–matrix interface properties [34]. Although the tensile modulus was calculated with the two methods, the values obtained with tensile tests are lower from those obtained with the cyclic procedure (Figure 3). Another important point of these tests is the difference between the slopes of the curve during the loading and unloading phases, the latter being more important (Figure 4). These phenomena may be explained by the fact that the matrix is subjected to crystallization during time. As shown through the FTIR analysis, the crystalline behavior of the composite slightly depends on the fibers content and morphology. Moreover, PHBHV is a semi-crystalline polymer, and changes in the microstructure of the polymer are possible at temperatures higher than the glass transition one. In our case, the tests were carried out at a temperature (23 °C) that is slightly higher than the glass transition temperature of the neat matrix (5 °C), as evaluated by Lorenzini et al. [54]. A possible mechanism occurring during the traction tests consists of different deformation steps. In particular, in the first stage of deformation, changes occur exclusively in amorphous zones that stretch. Successively, the crystalline areas start to slide parallel to the traction axis pulled from the stretched amorphous zones and, at the end, the highly stretched polymer chains align with the axis.

Since the polymer chains may vary during the loading, slopes in the loading and unloading phases can be different from each other. The crystallization phenomenon occurring during time was checked a posteriori using both mechanical and thermal tests. as showed in Appendix C.

### 4.2. Internal Morphology and Density of the Biocomposites

The morphology of the biocomposites can be perfectly identified by SEM images. Moreover, optical microscopy can provide important information on the effect of the processing on the fibers. Generally, when a compatibilizing agent is not used, fibers pull out from the matrix because of the poor adhesion between the two constituents. In the case of the biocomposites manufactured by extrusion and injection molding, a multi-layer effect along the cross section was also observed. This phenomenon was likely caused by the flow of melted material in the mold during the phase of injection molding and has a clear effect on the dispersion of the fibers in the matrix. These latter are mainly located in the upper part of the cross section while pure matrix is visible in the lower part (see Figure 5A,B). This effect can be explained as the result of low interfacial adhesion between the fibers and the matrix.

The fabrication procedure, from milling to injection molding, strongly impacts the length of the fibers, preserving their width (see Table 5). At low contents of reinforcement in the matrix, the fibers appear intact and long, while at high levels they are reduced to a finer powder. Keeping the rotational speed and time of mixing constant during the extrusion process, the mechanical torque of the extruder machine was observed to increase with the fiber content. As expected, after processing, there is a reduction in the aspect ratio due to the breakup of the fibers during processing. Moreover, the density of the fibers, and consequently that of the composites, is influenced by the degree of compression imposed during the step of injection.

### 4.3. Numerical Simulation

For the numerical simulation, a Mori–Tanaka model was adopted considering either cylindrical or spherical inclusions (see Section 2.4) and then implemented in a personal code (see Sansalone et al. [55] for details). The hypothesis of cylindrical fibers seems to be confirmed from MEB observations, while the hypothesis of a transversal isotropic organization seems to be plausible and suggested by the method adopted to realize the biocomposites. In particular, the injection molding procedure requires the injection of the extruded material into a mold along the vertical direction. Fibers and polymer chains are forced to enter into the mold and to basically align with the flow direction. Accordingly, both the Young modulus calculated using the ROM (see Equation (4)) and the E_3_ Young modulus provided by the Mori–Tanaka model with cylindrical inclusions correspond to the fiber direction. Although the transversely isotropic model with cylindrical inclusions is closer to the experimental results than the one with spherical inclusions (see Figure 8), it remains a relatively rough model, showing that the reality is different and more complex than assumed. The fibers are likely oriented in several directions, resulting in an anisotropic material. Moreover, the method used for manufacturing the materials causes a reduction in fiber size with effects on their shape, the latter not being perfectly cylindrical. As a first prospect, we could introduce damage effects in the model to take into account microcracking that occurs at the fiber-matrix interface. Another avenue of research to tackle the real geometry of the samples would consist of performing a direct finite element (FE) analysis using constructed geometries mimicking the composite, or grid built from 3D micro-CT images of the composite. Finally, if aiming at describing functionalized composites, a peculiar modelling of the force transmission trough the matrix/fiber interface would be necessary.

## 5. Conclusions and Perspectives

Biocomposites from Miscanthus giganteus fibers and PHBHV were fabricated using extrusion followed by injection molding. Tensile properties were evaluated using loading–unloading and simple traction tests. Young modulus increased slowly for low fiber contents, typically 5, 10, 20 wt %. This protocol could then easily be used for other type of green composites with other matrix and/or natural fibers. It is necessary to attain 30 wt % of fibers to observe a significant difference in the Young modulus. Although composites appear to be more rigid when compared to the pure matrix due to the presence of the reinforcement, they exhibit a decrease in the tensile strength. This effect can be justified by the lack of adhesion between the fibers and the matrix, which causes a loss of mechanical seal under tensile stress. An evidence of this lack of adhesion is that the fibers pull out from the composites instead of being totally immersed in the matrix, as shown by SEM images. Moreover, a difference in the Young modulus values calculated with the two methods (tensile tests and loading–unloading tests) was observed, indicating that PHBHV crystallize during time. This phenomenon was demonstrated by mechanical and thermal tests conducted a posteriori on the biocomposite with 5 wt % of raw fibers at different times.

The mechanical behavior of these materials was modeled by a two-phase Mori–Tanaka model where fibers were assumed either of cylindrical shape and oriented along the stress axis or spherical. The first model provides a better approximation of the experimental values of the Young Modulus, although, in reality, fibers are not perfectly cylindrical and oriented in one direction. Indeed, fiber size [55,56], shape [56], and orientation [55] may strongly affect the effective elastic properties of the composite and can explain the gap between the model and the experimental behavior. A better understanding of fibers orientation and shape via micro-CT images may be useful to implement a more accurate model. Moreover, the realization of PHBHV-based composites with different fibers size could be an interesting perspective in order to further improve the mechanical properties of these biocomposites. Lastly, the adhesion between fibers and matrix can be improved by the chemical modification of vegetal fibers or by a simple reactive extrusion [34].

## Figures and Tables

**Table 1:** Some of the extrusion and injection molding parameters. T_E_ extrusion temperature; n rotational speed; T_I_ injection temperature; T_m_ mold temperature.

T_E_ [°C]	n [tr.min^−1^]	T_I_ [°C]	T_m_ [°C]
160	60	165	45

**Table 2:** Technical data of Young Modulus and Poisson coefficient for *Miscanthus giganteus* and PHBHV. ^(a)^ Adapted from Kaack et al. [44] ^(b)^ Experimental value. ^(c)^ Assumed.

Constituents	Young Modulus E [GPa]	Poisson Coefficient [−]
Miscanthus *giganteus*	4.5 ^(a)^	0.3 ^(c)^
PHBHV	1.0 ^(b)^	0.3 ^(c)^

**Table 3:** Results of tensile tests on PHBHV/MIS composites considering different fiber content (0, 5, 10, 20, 30 wt %). The standard deviation values are also presented.

Samples	Tensile Modulus [MPa]	Tensile Strength [MPa]	Ultimate Strain [%]
PHBHV	889 ± 41	22.0 ± 0.48	9.9 ± 1.1
PHBHV_95_MIS_5_	1074 ± 44	17.0 ± 1.54	4.2 ± 0.7
PHBHV_90_MIS_10_	1238 ± 74	16.8 ± 1.67	4.4 ± 0.9
PHBHV_80_MIS_20_	1267 ± 90	15.8 ± 0.77	3.9 ± 0.2
PHBHV_70_MIS_30_	1891 ± 172	16.9 ± 1.03	3.3 ± 0.7

**Table 4:** Results of cyclic loading–unloading test on PHBHV/MIS composites at different fiber content (0, 5, 10, 20, 30 wt %). The standard deviation values are also presented.

Samples	Slope of Unloading Phase [N/mm]	Tensile Modulus [MPa]
PHBHV	115 ± 5.0	1012 ± 48
PHBHV_95_MIS_5_	134 ± 5.3	1185 ± 44
PHBHV_90_MIS_10_	159 ± 7.0	1408 ± 63
PHBHV_80_MIS_20_	196 ± 15.2	1736 ± 137
PHBHV_70_MIS_30_	228 ± 22.3	2032 ± 191

**Table 5:** Evaluation of fiber-size distribution. The standard deviation values are also presented.

Samples	Width, D (mm)	Length, L (mm)	L/D
PHBHV_95_MIS_5_	0.27 ± 0.04	1.72 ± 0.33	6.4
PHBHV_90_MIS_10_	0.20 ± 0.06	1.11 ± 0.44	5.6
PHBHV_80_MIS_20_	0.16 ± 0.01	0.82 ± 0.10	5.1
PHBHV_70_MIS_30_	0.18 ± 0.08	0.80 ± 0.09	4.6

**Table 6:** Density values for MIS fibers and composites materials calculated with weight values ^(a)^ and with a Helium pycnometer ^(b)^. The standard deviation values are also presented.

Samples	ν_MIS_n_	ν_MIS_m_	<ρ_MIS_> [g/cm^3^]	<ρ_C_calc_> [g/cm^3^]	<ρ_C_exp_> [g/cm^3^]
PHBHV	0	0	-	1.25^(a)^	1.083 ^(b)^
PHBHV_95_MIS_5_	5	4.4 ± 0.1	0.75 ± 0.03	1.214 ± 0.003 ^(a)^	1.233 ^(b)^
PHBHV_90_MIS_10_	10	7.9 ± 1.6	0.76 ± 0.05	1.187 ± 0.002 ^(a)^	1.176 ^(b)^
PHBHV_80_MIS_20_	20	17.5 ± 2.1	0.96 ± 0.01	1.186 ± 0.005 ^(a)^	1.184 ^(b)^
PHBHV_70_MIS_30_	30	27.1 ± 0.2	0.93 ± 0.03	1.143 ± 0.012 ^(a)^	1.240 ^(b)^

**Table 7:** Crystallinity parameters of PHBHV and its composites determined by FTIR-ATR analysis.

Sample	MIS Length [μm]	C_I_
PHBHV	-	1.07
PHBHV_95_MIS_5_	1000	1.06
PHBHV_95_MIS_5_	45	1.00
PHBHV_80_MIS_20_	1000	1.00

**Table 8:** R values obtained by FTIR-ATR analysis as function of DCP content for raw fibers of 1000 µm and for fibers extracted from biocomposites PHBHV_95_MIS_5_. The standard deviation values are also presented.

Sample	R
MIS	1.3 ± 0.01
PHBHV95MIS5	1.3 ± 0.01

**Figure A2:**
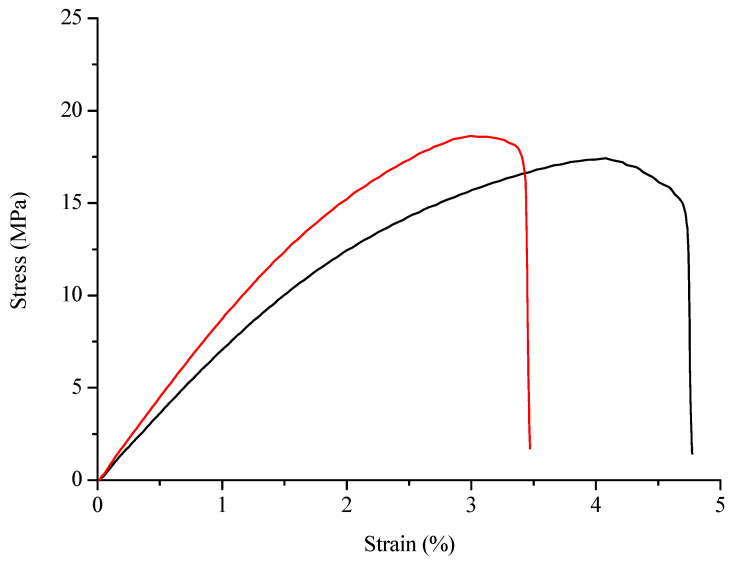
Strain-stress average curves for PHBHV_95_MIS_5_ composites tested after 8 days (black curve) and after 8 months (red curve).

**Figure A3:**
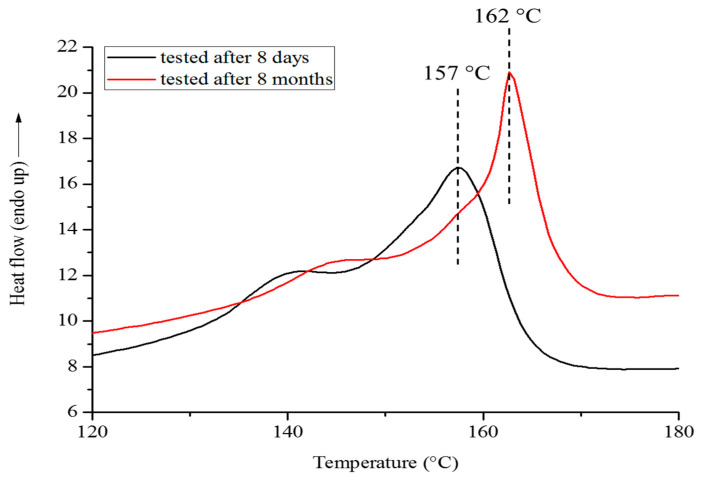
DSC first heating thermograms of PHBHV_95_MIS_5_ biocomposites tested after 8 days (black curve) and after 8 months (red curve).

## Data Availability

All the data can be obtained according to the LABEX MMCD policy.

## Appendix A. Identification of the Young Modulus of the Matrix

The Young modulus of the matrix was identified by inverse analysis which was then used as input data. Our raw input data are the slopes of the force/displacement curves of pure PHBHV samples in the unloading phase of the loading-unloading tests, see Table 4. The average value of these slopes, i.e., 115 N/mm, was assumed to be the stiffness of the PHBHV sample, K_s_.

A 2D Comsol model was then developed to model the part of the actual sample between the clamps of the testing machine, see Figure A1. The effective thickness of the model was set to that of the actual sample, i.e., H = 0.93 mm, the material was defined as linearly elastic and homogeneous, and simple traction conditions were applied on the left and right boundaries. A parametric analysis was then performed by letting the Young modulus of the material, E_m_, vary (while keeping fixed the Poisson ratio to 0.3), and the stiffness of the sample was computed as the ratio between the total force applied on the boundaries and the calculated elongation of the sample. The value of E_m_ best matching the experimental value was found to be about 1 GPa.

Since the numerical models developed afterward focus only on the “active” part of the sample, i.e., the central rectangular region sized L × B = 12 mm × 2 mm, a shape factor ʌ was calculated to transform the experimental values measured on the whole sample into values corresponding to its active part only. This shape factor is the ratio between the stiffness of the active sample, i.e.,

K_a_ = E_m_ × (B × H)/L (A1)

and the stiffness of the sample K_s_ (numerically computed). It turned out that

ʌ = K_a_/K_s_ = 1.37. (A2)

Assuming that this shape factor does not change when considering a composite sample, it allows computing the tensile modulus of composite material, E_c_, based on the experimental value of the stiffness of the composite sample, K_c_, namely:

E_c_ = ʌ × Kc × L/(B × H). (A3)

## Appendix B. Estimation of Volumetric Fraction of Fibers in the Specimens

The Mori–Tanaka method requires the knowledge of the volumetric fraction of the fibers in each composite specimen. This parameter $\phi_{F}$ = *V_F_/V_C_* is defined as the ratio between the volume of the fibers *V_F_* and the total volume of the composite *V_C_* = *V_F_* + *V_M_*, the volume of the matrix phase being *V_M_*. Note that we can measure the composite volume *V_C_* from the sample geometry. In order to obtain $\phi_{F}$ , we adopted an invasive procedure in order to separate the matrix from the fibers. First of all, every specimen manufactured by extrusion and injection molding was weighed and then solubilized under pressure and high temperature in 100 mL of dichloromethane with a rotation speed of 200 rpm for 30 min. After stirring, the solution was filtered and the solvent was evaporated in a static manner. Fibers were washed twice with the same procedure in order to eliminate the matrix attached. The matrix and the fibers were finally collected and their mass values $M_{M}$ and $M_{F}$ were obtained. Then, knowing the matrix density $\rho_{M}$ from the commercial provider, the fiber density $\rho_{F}$ , which may vary with processing due to the various degree of compression during the injection molding procedure, can finally be expressed as:

(A4)
ρF=MFVC−MMρM

Finally, the fiber volume *V_F_* = $M_{F}$ / $\rho_{F}$ can be deduced and thus the fiber volume content $\phi_{F}$ .

## Appendix C. Effect of Time on the Mechanical Properties of PHBHV-Based Composites

In order to evaluate the change in mechanical properties due to the time, traction tests were carried out on specimens of PHBHV_95_MIS_5_ after 8 days and then after 8 months. Results of these tests are showed in Figure A2. An increase in modulus and a decrease in the elongation at break are visible after 8 months, confirming that the crystallization phenomenon during time observed from other researchers is valid also for our composites. In a second time, we used differential scanning calorimetry (DSC) as an additional analysis to evaluate the crystalline behavior of the biocomposites; the results obtained are showed in Figure A3. A shift in the melt temperature from 157 °C to 162 °C and an increase in the crystallinity degree from 28% to 34% are visible after 8 months. This result is a further confirmation of the crystallization phenomenon.
